# Development of a robust SNP marker set for genotyping diverse gene bank collections of polyploid roses

**DOI:** 10.1186/s12870-024-05782-2

**Published:** 2024-11-14

**Authors:** Laurine Patzer, Tim Thomsen, David Wamhoff, Dietmar Frank Schulz, Marcus Linde, Thomas Debener

**Affiliations:** 1 Institute of Plant Genetics, Section Molecular Plant Breeding, Leibniz University Hannover, Hannover, Germany; 2 Institute of Horticultural Production Systems, Section Woody Plant and Propagation Physiology, Leibniz University Hannover, Hannover, Germany; 3 Current Address: Federal Office of Consumer Protection and Food Safety, Brunswick, Germany

**Keywords:** SNP, Fluorescence-based competitive allele-specific PCR, Genotyping, Gene bank management, Polyploid, Roses, Amplicon sequencing

## Abstract

**Background:**

Due to genetic depletion in nature, gene banks play a critical role in the long-term conservation of plant genetic resources and the provision of a wide range of plant genetic diversity for research and breeding programs. Genetic information on accessions facilitates gene bank management and can help to conserve limited resources and to identify taxonomic misclassifications or mislabelling. Here, we developed SNP markers for genotyping 4,187 mostly polyploid rose accessions from large rose collections, including the German Genebank for Roses.

**Results:**

We filtered SNP marker information from the RhWag68k Axiom SNP array using call rates, uniformity of the four allelic dosage groups and chromosomal position to improve genotyping efficiency. After conversion to individual PACE® markers and further filtering, we selected markers with high discriminatory power. These markers were used to analyse 4,187 accessions with a mean call rate of 91.4%. By combining two evaluation methods, the mean call rate was increased to 95.2%. Additionally, the robustness against the genotypic groups used for calling was evaluated, resulting in a final set of 18 markers. Analyses of 94 pairs of assumed duplicate accessions included as controls revealed unexpected differences for eight pairs, which were confirmed using SSR markers. After removing the duplicates and filtering for accessions that were robustly called with all 18 markers, 141 out of the 1,957 accessions showed unexpected identical marker profiles with at least one other accession in our PACE® and SSR analysis. Given the attractiveness of NGS technologies, 13 SNPs from the marker set were also analysed using amplicon sequencing, with 76% agreement observed between PACE® and amplicon markers.

**Conclusions:**

Although sampling error cannot be completely excluded, this is an indication that mislabelling occurs in rose collections and that molecular markers may be able to detect these cases. In future applications, our marker set could be used to develop a core reference set of representative accessions, and thus optimise the selection of gene bank accessions.

**Supplementary Information:**

The online version contains supplementary material available at 10.1186/s12870-024-05782-2.

## Background

The conservation of plant biodiversity is a crucial global issue. Genetic diversity is an important parameter for the maintenance of sustainable agriculture and horticulture, and is required for the creation of more productive and resistant plants [1]. Human intervention poses a severe threat to global biodiversity, particularly in light of climate change. Currently, the estimated number of plant species that are threatened by extinction has increased to 39.4% [2].

Due to genetic depletion in nature, gene banks play a critical role in the long-term conservation of plant genetic resources and the provision of a wide range of plant genetic diversity for research and breeding programs. There are approximately 1,750 gene banks worldwide that preserve more than seven million accessions of food crops [3]. In addition, there are approximately 2,700 botanic gardens [4] serving as gene banks for crops and ornamental plants that are important for both more sustainable breeding (e.g., resistance breeding) and for the preservation of cultural heritage. For this reason, the German Genebank for Roses was established in 2009, coordinated by the *Europa Rosarium Sangerhausen*. The world's largest rose collection currently comprises approximately 8,700 rose species and varieties (3,409 of which belong to the gene bank), which are available for breeding and research (G. Schulz, Europa Rosarium Sangerhausen, personal communication, April 29, 2024.). The availability of highly diverse genetic resources for roses is important, as only 8 to 10 of the 130 to 200 known wild rose species have contributed to more than 30,000 registered varieties [5–7]. Due to interspecific hybridisation and cross-fertilisation, the genomes of modern varieties are very complex, ranging from triploid (2n = 3x = 21) to octoploid (2n = 8x = 56) [8, 9]. However, tetraploid varieties (2n = 4x = 28) are mostly used for breeding [10].

For the efficient conservation of genetic resources in gene banks, the distinct identification of individual accessions is of the utmost importance. Gene bank collections may contain historical varieties with unknown breeding histories, leading to the cataloguing of varieties with synonymous or incorrect names. Confusion in labelling is also possible, particularly for vegetatively propagated plants like roses that have been in the field for years or need to be replaced due to damage. Among the 571 banana accessions from the gene bank of the CGIAR International *Musa* Germplasm Transit Centre that have undergone field verification, 10% were mislabelled, and an additional 6% were misclassified [11]. Using chloroplast markers, Zhang et al. (2021) [12] discovered that 17% of 53 rice seed accessions from gene banks or field collections were mislabelled. Therefore, it is crucial to develop effective methods for identifying and rectifying misclassifications in gene bank management. Often, authenticity verification is performed by documenting phenotypic data over many years. Because this procedure is strongly dependent on the experience of the observer as well as on the expression of phenotypic traits under different environments, the use of molecular genetic methods in combination with phenotypic data is crucial for improving gene bank management.

Molecular markers such as random amplified polymorphic DNA (RAPD), amplified fragment length polymorphism (AFLP), and simple sequence repeats (SSRs) are very useful for genetic diversity analysis [13]. However, it is difficult to automate and/or to score unambiguously these marker types and RAPDs are also difficult to reproduce (Jones et al., 1997). Therefore, high-throughput analyses and technology-independent genetic information that can be easily shared make single nucleotide polymorphisms (SNPs) the markers of choice [14]. Different techniques are available for SNP genotyping, such as PCR-based methods, genotyping by sequencing (GBS) and microarrays. For roses, the WagRhSNP chip with 68,893 markers was developed in 2015 and is based on sequence polymorphisms in expressed genes in garden and cut roses [15]. Due to the availability of high-quality rose genome sequences [16], these SNP data can be assigned to a position in the genome and used for genome wide association studies (GWAS) to identify markers of adventitious rooting, anthocyanin/carotenoid content and other floral traits [17–19]. However, SNP chips are very expensive if high genotype numbers are analysed with only a few markers.

PACE® and/or KASP™ markers are cost-effective alternatives for SNP detection in germplasm collections. These allele-specific PCR-based techniques can be easily scaled up to several thousand genotypes and to a flexible number of markers. In addition, they can be automated and display different ploidy levels. KASP™ and PACE® markers have already been used for variety identification in crops such as rice and faba bean [20, 21]. Additionally, Winfield et al. (2020) [22] developed a set of 21 KASP™ markers to distinguish 2,652 different apple varieties. To our knowledge, no reports on the development of PACE® or KASP™ marker sets for fingerprinting studies in garden roses have been published to date.

The aim of this study was to develop a robust, easy-to-use and cost-effective marker set that can be used to genetically characterise accessions in the German Genebank for Roses. For this purpose, SNPs with evenly distributed allele dosages and high calling rates were selected from the WagRhSNP chip, and PCR-based PACE® markers were developed and tested. The final marker set was tested for its suitability and resolution on 4,187 accessions, mainly from the gene bank stock. The results of different analytical approaches were compared, and five additional SSR markers were used to confirm suspected duplicates and genotypes with identical SNP profiles. In addition, 95 randomly selected genotypes were used to evaluate the additional information provided by amplicon sequencing and to examine an alternative SNP detection technology.

## Methods

### Plant material

A total of 4,187 individual rose accessions were sampled at the *Europa Rosarium Sangerhausen*, Germany (2974 cultivars and 127 species), and the *Bundessortenamt* (Federal Plant Variety Office) *Hannover*, Germany (1,086 cultivars). A list that assigns the identifiers to the origin is available under 10.6084/m9.figshare.25837849 and species names are listed in Additional File 1. Samples were collected in May/June 2021 and May 2022.

Young rose leaves that were not yet fully developed were sampled and stored at room temperature under dark and humid conditions to degrade polysaccharides. After two days, approximately 20 to 40 mg of leaf tissue was placed into the tubes of a 96-well rack and dried. On each rack, one tube was left empty to serve as a control. The remaining sampled leaf tissue was dried and stored as a backup sample.

### DNA extraction

Samples were isolated with the Mag-Bind® M1128 Plant DNA Plus Kit from Omega Bio-tek, Inc., using the Phoenix Pure 96 System (Hangzhou Allsheng Instruments Co., Ltd.). Deviating from the manufacturer's instructions for the kit, the incubation time was increased to 40 min after addition of the CSPL buffer. Furthermore, an additional sorbitol prewash step was performed according to Inglis et al. (2018) [23] to improve DNA quality.

The DNA quality and concentration were assessed using a NanoDrop™ 2000/2000c Spectrophotometer (Thermo Fisher Scientific Inc.) at 260/280 nm. For a set of arbitrarily selected samples, the quality and concentration were determined using agarose gel electrophoresis with lambda DNA as a standard. The DNA was then diluted with elution buffer/PCR-grade water to a concentration of 6 ng/µL and stored at -20 °C.

### PACE® genotyping assay

Two allele-specific forward primers and one common reverse primer were designed for each SNP based on the sequences 50 bp around the SNP using the PACE® Assay Design Template from 3CR Bioscience Ltd. The primer sequences can be found in Supplementary Table 1, Additional File 2. The PACE® assay was performed using PACE® 2.0 Genotyping Master Mix with a low Rox concentration according to the manufacturer's instructions. For a 384-well plate, 2.5 µL of DNA (15 ng) and 2.5 µL of master mix, including the assay mix, were combined in each reaction using the liquid handling system epMotion® 5075t from Eppendorf SE. Thermal cycling and fluorescent signal detection were conducted with a QuantStudio™ 6 Flex Real-Time PCR System (Thermo Fisher Scientific, Inc.). When using a newly designed assay mix, 26 to 39 PCR cycles were tested. To import the plate layout into the QuantStudio Real-Time PCR System (v1.3) software, an import file containing genotypes and markers was created using a custom-made Python script [24], which is available on request. Because the QuantStudio software used cannot score tetraploid organisms, the *FitTetra* package [25] from the statistical software* R* [26], version 4.0.4, was used for SNP dosage calling. Here, dip.filter = 0 and p.threshold = 0.95 were used instead of the default settings. The PACE® assay was repeated for DNA plates in which more than 20% of the genotypes could not be scored.

### Calculation of evenness

The evenness of allele dosage groups for each marker was calculated according to the following formula:

$$Evenness (I)= \frac{{H}_{S} }{\text{ln}(S)}$$

where *S* is the ploidy level and H_S_ is the Shannon‒Wiener index, which is calculated using the following formula:

$${Shannon-Wiener Index (H}_{S})=-\sum_{i=1}^{S}{p}_{i}*\text{log}{p}_{i}$$

where *p*_*i*_ is the relative frequency of the single allele dosage.

### Assessment of molecular identity

The genetic distance matrices were calculated using the *dist(method* = *'maximum')* function in *R* [26] based on the score files from *FitTetra* [25].

### Amplicon sequencing

Two PCRs were performed for amplicon sequencing. In the first PCR, gene-specific regions containing the SNP marker were amplified with M13-tailed primers. In the second PCR, unique sequence tags were added at the 5´ region of the primer binding to the M13 tail of the product from the first PCR. Gene-specific primers were designed using Primer3Plus [27]. The primers used for tagging were obtained from Pjevac et al. (2021) [28]. All primer sequences are provided in Supplementary Table 2 and Supplementary Table 3, Additional File 2.

PCR with gene-specific primers was performed in a 25-μL reaction containing 24 ng of DNA, 1 × Q5 buffer, 200 μM dNTPs, 0.5 μM forward and reverse primers and 0.02 U of Q5 High Fidelity DNA Polymerase (New England Biolabs GmbH). The second PCR with the tagging primers was also performed using Q5 High Fidelity DNA polymerase in a 50-μL reaction containing 2 μL of the PCR product from the first round. The cycling conditions were 98 °C for 30 s for initial denaturation; 25 (for the first PCR) and 12 (for the second PCR) cycles of 98 °C for 10 s, 60 °C (for the first PCR) and 69 °C (for the second PCR) for 20 s and 72 °C for 20 s; and a final extension of 2 min at 72 °C. Products from the second PCR were checked with agarose gel electrophoresis and pooled in equal amounts for each marker. The samples were purified using the NucleoSpin® Gel and PCR Clean-up Kit (MACHEREY–NAGEL GmbH & Co. KG) according to the manufacturer's instructions. The samples were diluted and sent to GENEWIZ Germany GmbH for Illumina sequencing.

Sequences were analysed using the AmpliSAT web tools [29], which are available at http://evobiolab.biol.amu.edu.pl/amplisat. AmpliCLEAN was used with a minimum Phred quality score of 30. The AmpliCHECK output was used for further analysis.

### Microsatellites (SSRs)

SSRs were amplified using the previously published primer pairs RMS043, RMS070, Rdr1, RMS066 and RMS059 [30–32]. Among these markers, Rdr1 tags a highly complex locus of TNL genes on rose chromosome 1. The primer sequences can be found in Supplementary Table 4, Additional File 2. Two multiplex reactions were performed using these primers. Multiplex reaction A included the pooled primer pairs RMS043 (IRD_700_ labelled) and RMS070 (IRD_800_ labelled). In multiplex reaction B, the primer pairs Rdr1 (IRD_700_ labelled), RMS066 (IRD_800_ labelled), and RMS059 (IRD_800_ labelled) were combined. The PCR reaction consisted of 20 µL and contained 18 ng of DNA, 2 µl 10 × Williams buffer (containing 100 mM Tris/HCl ph 8.3, 500 mM KCl, 20 mM MgCl_2_, 0.01% gelatine), 200 µM dNTPs, 0.25 µM each of the forward and reverse primers, and 0.05 U/µL DCS polymerase. The PCR conditions were as follows: initial denaturation for 5 min at 94 °C, followed by 25 cycles of 30 s at 94 °C, 30 s at 56 °C and 30 s at 72 °C, and a final step at 72 °C for 5 min. Five microlitres of PCR product was diluted with 50 µL formamide and then incubated at 95 °C for 5 min. SSR marker bands were visually scored on a 6% polyacrylamide gel using vertical electrophoresis and a LI-COR 4200 DNA Analyser (LI-COR, Inc.) as described in Spiller et al. (2011) [31].

## Results

### Selection of the marker set based on chip data

Data generated using the 68 k WagRhSNPChip from previous studies [19] were used to determine the allele dosage of an association panel consisting of 95 garden rose cultivars. The 68 k WagRhSNPChip uses two specific probes, called the 'first probe' and 'second probe', to detect genetic variations. The probes bind on opposite sides of the SNP, providing two sets of fluorescence data. Using FitTetra, 37,514 of the 68 k SNPs on the chip were scored for probe 1 and 38,925 for probe 2, resulting in a total of 47,691 unique SNPs. To identify markers with a uniform allele distribution across the panel as well as a high calling rate, the markers were filtered based on the following criteria: 1) at least 10% of the genotypes are in each of the five possible allele dosage clusters, 2) 90% of all genotypes are assigned with a 99% probability to an allele dosage group, and 3) the average of the maximum probability of all genotypes belonging to any allele dosage group is 99%. This resulted in 189 markers of potential interest, 40 of which were filtered from both probes. Based on the allele distribution and calling rates, the markers were converted to PACE® markers and tested on 95 to 190 accessions until 20 of the 75 converted PACE markers could be selected for genotyping. The marker combination was selected in such a way that at least two markers were distributed on each of the seven chromosomes (Supplementary Fig. 1, Additional File 3).

### Allele dosage distribution, calling rate and robustness of the marker set analysed in 4,187 rose accessions by comparing different plate layouts

**Fig. 1:**
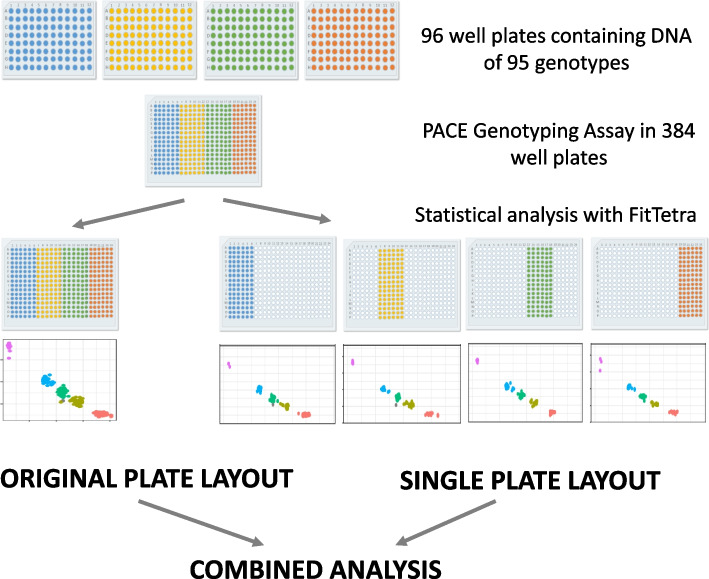
Determination of marker quality by combining different layouts. The DNA from 95 genotypes and one negative control was stored in 96-well plates. For the PACE genotyping assay, DNA from two to four plates was pipetted into 384-well plates. The statistical analysis was conducted with FitTetra using two different approaches. First, the original plate layout was used for the analysis. Second, the fluorescence data from the 384-well plate were subdivided into 96 samples stored on one DNA plate (single plate layout). For each of the subsets, an independent analysis was conducted. Then, the results of both approaches were compared (combined analysis)

For fluorescence-based detection of allele expression in a biallelic SNP, the allele dosage can only be classified in comparison to the fluorescence ratios of the other samples in the particular analysis. Consequently, the composition of samples in an analysis unit can have an effect on calling. Therefore, a total of 4,187 accessions were genotyped with the developed marker set of 20 PACE® markers, and fluorescence data were analysed with *FitTetra* using two different approaches (Fig. 1). In the first approach (the original plate layout), the data were used as they were placed in the 384-well plate layout. In general, 380 genotypes and four negative controls were analysed with one marker or 190 genotypes, and two negative controls were analysed with two markers per plate. Thus, 190 to 380 genotypes per marker were combined in one analysis. In the second approach (the single plate layout) the 190 to 380 genotypes on one 384-well plate were divided into groups of 95 genotypes according to the original arrangement on the 96 well DNA plates and then analysed separately. Additionally, both approaches were combined by using both scores and then compared with each other. Adding fluorescence values from separate plate runs was avoided, as different runs may slightly differ in fluorescence properties and therefore add additional noise to the analysis.

The proportion of genotypes that were unambiguously assigned to an allele dosage with a p-value of less than 0.05 was on average 91.4% for the original plate layout, 93.6% for the single plate layout, and 95.2% for the combination of the two approaches (Table 1). The best marker for the single plate layout, RhK5_5648_324, scored 97.1% for all genotypes and was also the best marker for the original plate layout, with a calling rate of 96.3%. After combining the scores from the original plate layout and single plate layout, the calling rate increased to 97.3%. The highest calling rate after combining the two approaches was obtained for the marker Rh12GR_60359_1361 (97.9%). Before combining the two approaches, these calling rates were only 95.2% (single plate layout) and 94.8% (original plate layout). The two markers with the lowest calling rates were RhK5_125_737 (89.8% after combination) and RhMCRND_17396_291 (87.3% after combination). All other markers called at least for 94% of the genotypes after combining the two approaches.

**Table 1:** Proportion of called genotypes in the panel of 4,187 rose genotypes per plate layout

Marker	Called genotypes from single plate layout [%]	Called genotypes from original plate layout [%]	Called genotypes after combination [%]
RhMCRND_17396_291	86.3	81.1	87.3
RhK5_125_737	79.5	78.7	89.8
RhK5_11434_711	92.0	89.3	94.1
RhK5_1295_1946	93.5	90.1	94.5
RhK5_6968_582	94.0	91.7	94.7
RhK5_69_1627	94.8	94.1	95.1
Rh12GR_2923_1285	93.8	93.5	95.2
RhMCRND_6703_1113	94.5	92.8	95.2
Rh12GR_7355_525	94.8	89.8	95.7
RhK5_11100_1011	94.9	94.9	95.8
RhMCRND_11585_178	94.8	90.7	95.8
RhK5_299_2775	94.0	87.9	96.0
RhK5_11411_1602	95.8	93.5	96.3
RhK5_978_1131	96.0	95.6	96.3
RhK5_10792_6318	94.1	94.3	96.8
RhK5_1804_1724	96.4	96.3	96.8
Rh12GR_10867_316	96.3	92.8	97.0
RhK5_8422_105	94.3	90.3	97.1
RhK5_5648_324	97.1	96.3	97.3
Rh12GR_60359_1361	95.2	94.8	97.9

The table is sorted in ascending order for the column “Called genotypes after combination [%]”

The combination of markers with an even distribution of allele dosages has greater discrimination potential than markers with strongly biased distributions. To assess whether the allele dosages were equally distributed, the proportion of the genotypes within a cluster was considered, and the evenness was calculated based on the Shannon‒Wiener index. In the single plate layout, the allele dosages of the genotypes were equally distributed among all five possible clusters (P0, P1, P2, P3, and P4) for the markers RhMCRND_17396_291 and RhK5_11100_1011, with evenness values of 0.996 and 0.988, respectively (Table 2). In contrast, the RhK5_8422_105 marker had the lowest evenness value of 0.740. Although 59% of the genotypes had allele dosage four, all other allele dosages were underrepresented, with proportions ranging from 3 to 16% (Table 2).

**Table 2:** Allele distribution across the five clusters per marker for the single plate layout (4,187 genotypes)

Marker	P0	P1	P2	P3	P4	Evenness
RhK5_8422_105	0.03	0.13	0.16	0.09	0.59	0.740
RhK5_6968_582	0.35	0.34	0.21	0.07	0.03	0.841
Rh12GR_10867_316	0.30	0.33	0.26	0.09	0.02	0.851
Rh12GR_60359_1361	0.03	0.13	0.25	0.28	0.32	0.887
Rh12GR_2923_1285	0.24	0.26	0.29	0.17	0.03	0.913
Rh12GR_7355_525	0.19	0.28	0.32	0.14	0.07	0.935
RhMCRND_11585_178	0.06	0.20	0.24	0.23	0.27	0.945
RhK5_69_1627P	0.09	0.23	0.33	0.22	0.14	0.947
RhK5_5648_324	0.20	0.28	0.29	0.13	0.10	0.949
RhK5_10792_6318	0.23	0.28	0.27	0.13	0.09	0.949
RhK5_11434_711	0.30	0.24	0.20	0.10	0.16	0.963
RhK5_978_1131	0.18	0.20	0.29	0.24	0.10	0.966
RhMCRND_6703_1113	0.22	0.18	0.26	0.24	0.10	0.970
RhK5_1804_1724	0.17	0.20	0.30	0.21	0.11	0.971
RhK5_299_2775	0.19	0.28	0.24	0.15	0.13	0.974
RhK5_1295_1946	0.15	0.15	0.26	0.27	0.17	0.978
RhK5_11411_1602	0.18	0.15	0.23	0.29	0.15	0.978
RhK5_125_737	0.14	0.21	0.27	0.23	0.14	0.979
RhK5_11100_1011	0.13	0.19	0.23	0.21	0.24	0.988
RhMCRND_17396_291	0.24	0.19	0.21	0.19	0.17	0.996

P0 = Allele dosage 0; P1 = Allele dosage 1; P2 = Allele dosage 2; P3 = Allele dosage 3; P4 = Allele dosage 4. The evenness was calculated based on the Shannon‒Wiener index. The value for evenness ranges between 0 (completely unequal distribution of individuals among the individual species) and 1 (totally equal distribution)

**Fig. 2:**
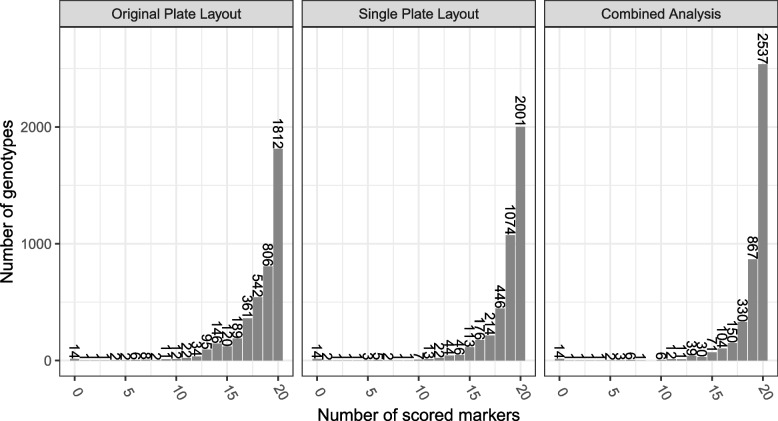
Number of genotypes with scored markers compared among different scoring approaches. The calculations were performed for the single plate layout, original plate layout and the combination of the scores from both layouts. The total number of all genotypes was 4,187

Using the two different scoring methods, the original and the single plate layout, the number of genotypes scored with all 20 markers could be increased from 1,812 to 2,537 of all 4,187 accessions (Fig. 2). Fourteen genotypes could not scored with any of the markers and analysis methods.

The robustness of the marker scoring was assessed by comparing the scores from the single plate layout analysis and the original plate layout analysis. For two markers no differences in scores were noted, maximum error rate of 5% was observed for 13 additional markers (Table 3). Two markers had differences in scores greater than 10%. The mean error rate of all markers was 3%. For all markers, the differences were mainly in only one difference in the allele dosage group (e.g. ABBB or AAAB instead of AABB). With the exception of marker RhK5_125_737, the proportion of differences in more than one allele dose group was less than 1%.

**Table 3:** Differences in the scores between single plate layout and original plate layout using 4,187 genotypes

Marker	Proportion of differences [%]	Differences in 1 allele dosage group [%]	Differences in 2 allele dosage groups [%]	Differences in 3 allele dosage groups [%]	Differences in 4 allele dosage groups [%]
RhK5_1804_1724	0				
RhK5_69_1627	0				
RhK5_978_1131	0				
Rh12GR_10867_316	1.1	1.1			
RhK5_5648_324	1.1	1.1			
RhMCRND_17396_291	1.1	0.4	0.7		
RhK5_6968_582	1.5	1.5			
RhK5_11411_1602	1.6	1.6			
RhK5_10792_6318	1.7	0.8	0.5	0.4	
Rh12GR_2923_1285	1.8	1.8			
RhMCRND_11585_178	2.1	2.1			
RhK5_11434_711	2.3	2.3			
RhK5_1295_1946	2.3	2.3			
Rh12GR_7355_525	2.7	2.7			
Rh12GR_60359_1361	2.8	2.3	0.5		
RhK5_11100_1011	3.1	1.8	0.6	0.1	0.6
RhMCRND_6703_1113	5.4	5.4			
RhK5_299_2775	6.0	6.0			
RhK5_8422_105	13.6	13.5	0.1		
RhK5_125_737	18.0	10.4	3.2	4.4	

### Validation of the suspected duplicates

**Fig. 3:**
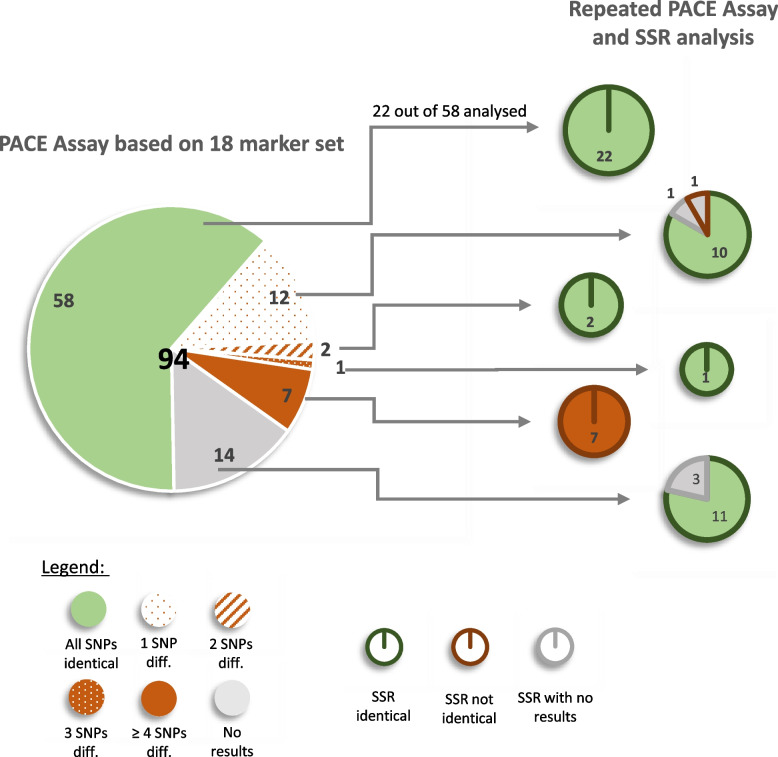
Analysis of 94 assumed genotype pairs using the 18-PACE marker set and additional SSR analyses. First, the PACE assay was performed on 4,187 accessions, after which the scores of the pairs were compared. To confirm the results, the DNA was isolated again, and the PACE assay was repeated. Additionally, SSR marker analysis with five markers was performed. “No results” means that more than four of the 18 SNPs or more than three of the five SSR markers failed

Among the 4,187 accessions, 94 cultivars occurred twice in the collections (once at each of the locations). The molecular identity of these 94 pairs was checked by PACE® genotyping assays. The markers RhK5_125_737 and RhK5_8422_105 were excluded from this analysis due to differences in scores of more than 10% between different plate layout analyses (Table 3), resulting in a core set of 18 robust markers. Overall, 58 of the 94 pairs (62%) had the same SNP pattern (Fig. 3). Fifteen of the pairs differed in one to three SNPs, and seven of the hypothetical duplicates had differences in more than three SNPs. For 14 pairs, more than four markers could not be scored, so these were excluded from the statistical analysis.

To confirm these results, the genomic DNA of 116 of the accessions (58 pairs) was isolated again, and the PACE® genotyping assays were repeated. This included all pairs in which a difference of at least one SNP was detected or in which no conclusion could be drawn due to failures in more than four SNPs. In addition to these 36 pairs, 22 randomly selected duplicate pairs with no marker differences from the first set of DNA samples were included. All putative pairs were arranged in the repetition in such a manner that the pairs were located on the same plate. In addition, SSR analysis with five markers was conducted for the 116 samples.

The 22 pairs in which no differences were initially found in the first set of samples were confirmed using the repeated PACE® assay and the SSR analyses (Fig. 3). Additionally, all seven pairs with differences in more than three SNPs in the first PACE® assay also had different marker patterns in the repeated PACE® assay and the SSR analysis. The percentage of differently called markers in the first and second PACE® assay was approximately 1.3%. The 19 markers scored differently between the first and second PACE assay were present in 15 accessions. Eleven of these cases showed deviations in one allele dosage group, three cases showed deviations in two allele dosage groups and five cases showed a non-scored marker (Additional File 4). For the pairs that could not be analysed in the first PACE® assay due to too many failures, eleven of the 14 pairs in the repeated PACE® assay showed no difference in the SNPs between all pairs, which was also confirmed with the SSR analysis. Three of the pairs still had more than four missing markers. The results of the repeated PACE® assay based on the 18-marker set and the SSR analysis were consistent for all pairs.

### Number of other accessions with identical SNP profiles

**Fig. 4:**
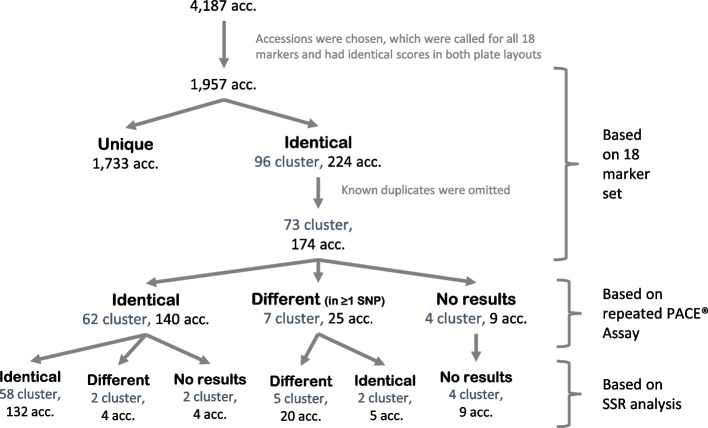
Number of accessions with identical SNP profiles. A total of 4,187 accessions (acc.) were analysed with 18 PACE markers and then filtered to exclude accessions with missing values and non-identical scores in both plate layouts. This resulted in 1,957 accessions, of which 1,733 had a unique SNP profile. The remaining 224 accessions clustered in 96 groups. After excluding known duplicates, 174 accessions in 73 clusters remained. Additional SSR analysis was performed, and the PACE assay was repeated based on newly isolated DNA

To assess the discriminatory power of the 18-marker set, the number of unique SNP profiles was determined. For this purpose, only accessions in which all 18 markers were called without any missing values and in which the scores were identical between the single plate layout and original plate layout (proof of concept) were analysed. Of the 4,187 samples, 1,957 accessions fulfilled these requirements. All but 224 accessions showed a unique SNP profile according to the 18-marker set. These 224 accessions were grouped into 96 different clusters, each of which had the same SNP profiles (Fig. 4). Subtracting the known and verified duplicate accessions described in the previous chapter, resulted in 174 accessions grouped in 73 clusters. The clusters often consisted of two or three accessions. Interestingly, one cluster consisted of 11 accessions.

To confirm the accessions with identical PACE® marker profiles, SSR analyses with five markers were conducted, and the PACE® genotyping assay based on the 18-marker set was repeated. For the repetition, the DNAs from individuals of the same cluster were located on the same 384-well plate. Using this method, the molecular identity of 62 clusters, containing 140 accessions, out of the 73 clusters was confirmed. Of these 62 clusters, 58 clusters were further confirmed with the five SSR markers, and two groups showed no band patterns. However, two clusters with two accessions each, which were identical according to both PACE® assays, were found to exhibit differences in the band pattern for two of the five SSR markers or for all five markers respectively.

In seven of the 73 clusters, the repeated PACE® genotyping assay showed differences between the individuals, which were confirmed in five clusters using the SSR markers. In four of the five groups, the PACE® assay showed differences in one to two SNPs. However, in one case, differences in all 18 SNPs were detected, indicating that the sample was mixed up. No conclusion could be drawn for four clusters because the SSR markers did not produce fragments or the samples were no longer available.

### Comparison of the performance of the marker set between the PACE® genotyping assay and amplicon sequencing data

PACE® genotyping assays are only one method for determining the allele configurations of SNPs, and direct sequencing with NGS platforms represents an alternative even for large numbers of genotypes. Therefore, primers were successfully designed for 13 SNPs out of the 20-marker set and used to analyse 95 out of the 4,187 accessions randomly selected by amplicon sequencing to compare the information content as well as the workload of the PACE® assays. After excluding genotypes with fewer than 100 reads and sequences with less than 10% of the total reads of the genotype, the results were compared with the PACE® genotyping results using *AmpliSat* and the *R* package *FitTetra* as allele dosages. For eight of the 13 markers, differences in the allele dosage estimations between the PACE® assay and amplicon sequencing were observed for a maximum of four genotypes (Table 4). For the markers RhK5_10792_6318 and RhK5_1295_1946, 18 to 38 accessions were called differently. To exclude sequencing errors and errors due to contamination or sample handling, amplicon sequencing was repeated for the markers RhK5_10792_6318 and RhK5_1295_1946. For RhK5_10792_6318, 25 genotypes were differently called also in the replicate. The marker RhK5_1295_1946 could not be successfully called by FitTetra in the repetition. Interestingly, five genotypes showed differences in the repeated amplicon scoring for the marker RhK5_10792_6318, which had previously been correctly assigned. The markers RhK5_8422_105 and RhMCRND_11585_178 could not be called using FitTetra because there were too few genotypes with more than 100 reads. The analysis with genotypes above 50 reads showed that the marker RhK5_8422_105 showed differences in scoring between PACE assay and Amplicon in 22 of the genotypes and the marker RhMCRND_11585_178 in only one of the genotypes (Additional File 5).

**Table 4:** Comparison between PACE® and amplicon scoring within the same SNP analysed in 95 genotypes

Marker	Number of genotypes with identical scoring	Number of genotypes with differences in scoring	Number of unscored genotypes in total	Number of unscored genotypes in the PACE assay	Number of unscored genotypes in the amplicon analysis
Rh12GR_2923_1285	79	0	16	5	14
RhK5_5648_324	79	0	16	1	16
RhK5_6968_582	73	1	21	5	18
RhK5_978_1131	85	1	9	3	9
RhK5_11411_1602	83	2	10	2	9
RhMCRND_6703_1113	77	2	16	13	6
Rh12GR_7355_525	59	3	33	13	26
RhK5_299_2775	69	4	22	2	21
RhK5_69_1627	62	9	24	6	22
RhK5_10792_6318	58 (50)	18 (25)	19 (20)	1	19 (20)
RhK5_1295_1946	30 (NA)	38 (NA)	27 (95)	5	25 (95)
RhK5_8422_105	NA	NA	95	3	95
RhMCRND_11585_178	NA	NA	95	4	95

The numbers in brackets are the numbers in the repetition

### Discrimination accuracy of the markers from the marker set using the amplicon sequencing approach

The amplicon sequencing primers were designed so that the amplicons excluding the tags had lengths between 177 and 308 bp (Table 5). The sequence information was used to further identify polymorphisms located around the actual SNP in the 95 randomly selected genotypes. Two of the 95 genotypes had fewer than 100 reads for all 13 markers and were therefore excluded from further analyses. The marker Rh12GR_2923_1285 had the lowest number of polymorphic sites and different haplotypes, detecting eight different haplotypes and distinguishing 24 different genotypes based on six SNPs (Table 5, Additional File 6, and Additional File 7). The marker RhK5_8422_105, with the largest number of polymorphisms (56 polymorphic sites and 27 haplotypes) in a 177-bp product, was excluded from further analysis because more haplotypes per genotype were distinguished than expected after filtering and unspecific primer binding sites were found. The marker RhK5_978_1131 could distinguish the largest number of genotypes (65) with 24 polymorphic sites. The number of actual genotypes resulting from the possible combinations of haplotypes of a marker varied from 18 (RhK5_69_1627) to 65 (RhK5_978_1131).

**Table 5:** Number of variants for each marker according to amplicon sequencing in 95 genotypes

Marker	Amplicon size without tags [bp]	Number of genotypes with reads > ≥ 100	Number of polymorphic sites	Number of haplotypes	Number of distinguishable genotypes
Rh12GR_2923_1285	245	84	6	8	24
RhMCRND_6703_1113	211	89	7	9	26
RhK5_69_1627	230	75	10	9	18
RhK5_1295_1946	239	73 (57)	13 (14)	10 (11)	21 (18)
RhK5_10792_6318	308	81 (82)	14 (14)	10 (10)	52 (55)
RhK5_11411_1602	304	86	16	13	45
RhK5_299_2775	241	80	16	15	42
Rh12GR_7355_525	233	72	17	14	52
RhK5_6968_582	302	80	21	21	54
RhMCRND_11585_178	237	45	24	20	42
RhK5_978_1131	303	92	24	16	65
RhK5_5648_324	301	81	24	17	63
RhK5_8422_105	177	64	56	27	n.a

The two genotypes for which fewer than 100 reads were assigned for any of the markers were excluded from the analysis. Regarding the number of genotypes, tetraploidy was assumed. The polymorphic sites included SNPs and INDELs. The table is sorted in ascending order of the number of polymorphic sites. n.a. = not available. The numbers in brackets are the numbers in the repetition

When combining only two different amplicon markers, the number of actual genotypes that could be distinguished increased to 47 (RhK5_69_1627 and RhK5_1295_1946 combined) up to 88 (RhK5_978_1131 and RhK5_5648_324) out of the 93 possible combinations (Table 6). On average, 76 of the 93 genotypes could be distinguished when two amplicon markers were combined. When all markers were used, all of the 93 genotypes could be distinguished.

**Table 6:** Number of actual variants after combining the markers from amplicon sequencing of the 95 genotypes

	**_1113**	**_1131**	**_1285**	**_1602**	**_1627**	**_178**	**_1946**	**_2775**	**_324**	**_525**	**_582**	**_6318**
_1113	26	78	57	72	55	61	56	76	81	75	75	79
_1131	78	65	76	85	83	78	80	85	88	87	87	86
_1285	57	76	24	71	54	58	49	70	75	74	74	77
_1602	72	85	71	45	71	70	72	86	82	82	84	84
_1627	55	83	54	71	18	52	47	71	79	75	71	73
_178	61	78	58	70	52	42	53	68	74	72	71	69
_1946	56	80	49	72	47	53	21	64	76	73	67	70
_2775	76	85	70	86	71	68	64	42	86	80	81	83
_324	81	88	75	82	79	74	76	86	63	85	84	81
_525	75	87	74	82	75	72	73	80	85	52	81	83
_582	75	87	74	84	71	71	67	81	84	81	54	82
_6318	79	86	77	84	73	69	70	83	81	83	82	52

The number of haplotypes identified when using the marker alone are marked in yellow. The marker names have been shortened to the last numbers after the underscore, e.g., RhMCRND_6703_1113 = _1113

## Discussion

In the present study, a set of 18 PACE® markers was developed based on SNP chip data and used to analyse 4,187 accessions, mainly from the German Genebank for Roses. The purpose of this marker set is to uniquely characterise the accessions to identify duplicates and to detect mislabelling in large rose collections. In the future, molecular information could also be used to calculate genetic distances and assess genetic diversity in collections. According to UPOV, the requirements for markers used for DNA profiling include high discriminatory power, reproducibility within and between different laboratories, and the possibility of varying the number of samples and/or the number of markers [33]. Therefore, we decided to use SNP markers in our study because they exhibit the following advantages over other marker types: 1) SNP markers can be automated, 2) the biallelic format is easy to process, 3) dosage information can be obtained for polyploids, and 4) these markers can be monitored on different platforms.

### Considering how many markers are needed

The number of markers included in the genotyping set is a trade-off between cost/time and resolution. Assuming that up to five different SNP configurations of a biallelic marker can exist for tetraploid organisms (AAAA, AAAT, AATT, ATTT, and TTTT), the number of markers required to distinguish 4,187 genotypes is six (Supplementary Fig. 2, Additional File 3). Thus, 18 markers could theoretically discriminate ${5}^{18}$ accessions. For this calculation, an ideal marker set was assumed, consisting of markers that are completely independent of each other, all having a uniform allele distribution and an error-free calling rate of 100%. In practice, however, these assumptions are not fulfilled. The calling rate we achieved (95.2%) was greater than that of Tang et al. (2022) [34] who used 48 KASP™ markers from the LGC Genomics database to differentiate 518 rice varieties with a calling rate of 92.69%. Nevertheless, a 100% calling rate cannot be achieved for various reasons. Primers may not bind due to low DNA quality or rare variants. In addition, the allele dosage clusters may not be sufficiently separated; thus, some genotypes cannot be assigned with sufficient certainty. We analysed all accessions with FitTetra under the assumption that the accessions were tetraploids (4x = 28). However, cultivated roses can also be triploid (3x = 21), and wild species are often diploid (2x = 14) [35]. Distinguishing diploid and tetraploid yam accessions, Cormier et al. (2019) [36] reported good allele dosage assessment quality when using KASP™ assays and the five allele dosage clusters of tetraploids. But although diploid organisms are expected to show the same homozygous and balanced allele dosage ratios as tetraploids (0:2 = 0:4; 1:1 = 2:2; 2:0 = 4:0), triploid roses could show clusters between the expected allele dosage ratios (1:3; 3:1) and are therefore not called. Including ploidy level in the statistical evaluation may improve the calling rate, but analysing each sample with a flow cytometer would lead to much higher costs and considerably more personnel effort. Therefore, the calling rate in our study (95.2%) together with our evaluation of the robustness of the markers is a good compromise for a cost-effective marker assay with sufficiently high precision, even if ploidie levels are mixed in the samples.

In addition, the allele distribution in our study was not uniform. Although we only selected markers in the SNP chip where at least 10% of the 95 genotypes were scored in each of the allele dosage groups, the minor frequencies of the genotypes in the allele dosage groups in the 4,187 accessions screened by PACE assay often had significantly lower values (2–17%). We assume that the differences are because the population structure of the genotypes in the WagRhSNP chip data is not representative. Since roses are highly polymorphic and heterozygous, there may be bias in some SNPs of the chip, which is designed based on a small panel of 95 genotypes that will not cover other genotypic groups properly (ascertainment bias) [37, 38]. This feature also likely explains why only 20 out of the 75 markers converted from the SNP chip analysis were selected. This conversion rate of 27% is significantly lower than that reported in other studies. Shen et al. (2021) [39] were able to use 69.4% of their KASP™ markers for high-quality genotype clusters in broccoli, whereas Winfield et al. (2020) [22] showed that 21 out of 31 (68%) selected KASP™ markers had a high level of reproducibility and good discrimination capacity in apple varieties. However, both Winfield et al. (2020) [22] and Shen et al. (2021) [39] scored only diploid organisms and did not expect tetraploid clusters. Due to the low transformation rate of the markers in our study, we preferred to test the performance of individual markers first; however, many approaches are available to program a pipeline in advance to test marker combinations in silico [22, 40, 41].

Given that the assumptions of a 100% call rate and a uniform allele distribution are not met in reality, we tested three-fold more markers (18 markers) than required under these theoretical assumptions. We showed that the resolution of our marker set was comparable to that of the SSR markers. Only four out of the 1,957 accessions (0.2%) could not be distinguished with our developed marker set with 18 PACE® markers (including repetitions), but could be distinguished with SSR markers.

### Robustness of the developed marker set

Assessing the robustness of the marker set is crucial, particularly when comparing numerous accessions that cannot be scored in a single analysis unit. The evaluation of the PACE® assays is not possible in relation to the individual sample; this can only be achieved by comparing the fluorescence ratios with other samples on the analysed plate. By comparing the scores of different plate layouts, we investigated the influence of the composition or number of samples on the scoring of the respective marker. In our study, the markers had an average disagreement of 3% between the different analysis units (the single plate layout and the original plate layout). Two markers, RhK5_8422_105 and RhK5_125_737, showed differences between the analysis units in more than 10% of all analysed accessions and were therefore excluded from the marker set. These deviations may be due to underrepresentation of an allele dosage in the population analysed. The RhK5_8422_105 marker had the lowest evenness with an allele dosage in which only 3% of all accessions occured. For the second poorly performing marker, RhK5_125_737, the calculated evenness of 0.979 initially seems relatively high; however, the evenness is clearly distorted by incorrect calling by FitTetra (Additional File 8). Therefore, it is not sufficient to only consider evenness. Our study demonstrates the importance of conducting robustness analyses for newly developed markers, particularly when comparing samples that are not located on the same plate and therefore not assessed in the same analysis unit. Some confirmed duplicate accessions had differences in one to three SNPs when analysed using the two different plate layouts. When comparing pairs on the same plate, the SSR and PACE® results were identical. This suggests the need to establish an error threshold to account for statistical uncertainties when analysing accessions on different analysis units, even when markers are carefully selected. In particular, samples that only differ in one allele dosage group for one SNP should be re-analysed with several markers or on the same 96 or 384 well plate, which can be statistically evaluated as one analysis unit. Evaluation of marker quality by comparing the scores of genotypes in relation to two differently sized groups was not found in the current literature on fingerprinting studies. In the published studies, the error rate was often determined by comparing a set of technical replicates or was not performed at all [21, 22, 36, 39, 42]. Therefore, we tested a new approach by analysing different plate layouts. However, our method involved subsampling, and the genotypes on the plate were not completely rearranged. It would be interesting to analyse a larger unit with 1,536 samples on one plate (for which a high-throughput cycler is required) and then subsample hundreds of times to evaluate the robustness of a marker based on the allele dosages of the accessions localised on the same plate.

### Amplicons have a significantly higher resolution, but PACE® assays are currently more cost-effective, and the platform change is not trivial

SNPs can be detected using various techniques. As NGS technologies continue to evolve, precision and output constantly increase, whereas the cost per sequenced base decreases [43]. Consequently, amplicon sequencing potentially represents the first application of NGS to address the problems of systematic SNP genotyping in roses. In our study, we compared amplicons with PACE® markers for a small set of genotypes in terms of cost and resolution. For the amplicon sequencing of 95 genotypes, we spent approximately 380 € of consumables and sequencing (mainly for the proof reading Taq polymerase) per marker, whereas PACE® genotyping cost approximately 7 € for 95 genotypes (DNA isolation not included). Additionally, for amplicon sequencing, the workload is much greater given the need for additional steps in addition to the two PCRs for pooling and purification of the products. Nevertheless, there is no need to purchase and maintain a real-time PCR system. Furthermore, although the PACE® assays only provide information on the respective SNP, amplicon sequencing provides additional sequence information around the SNP. In our study, the amplicon sequences showed between six to 24 SNPs and/or INDELs compared to one bi-allelic SNP in the PACE® assay. Therefore, NGS technologies such as amplicon sequencing might use fewer loci in total to unambiguously characterise accessions of roses in the future, and costs and labour can be further reduced by using a multiplex PCR approach like it is already done in rice, soybean and other major agricultural crops or for pathogen detection [44–46].

However, the desired platform change for the detection of SNPs is not easily implemented. In our study, we investigated the comparability of SNP allele dosages between PACE® technology and amplicon sequencing. The agreement between PACE® and the amplicon markers for the analysed SNPs was only 76% When comparing SeqSNP and KASP™, agreement rates of 94% and greater have been reported in the literature [22]. One explanation for the low agreement rate is that too few samples were scanned, the proportion of incorrectly called samples was only 4%. In order to further increase the agreement rates and the general statistical certainty, it would be advisable to set the read threshold per genotype well above 100. Because the determined allele dosages within the investigated SNP differed strongly between the PACE® analyses and the amplicon sequencing data for the markers RhK5_8422_105, RhK5_1295_1946 and RhK5_10792_6318, the primer binding sites for the PACE® primers were examined on the basis of the sequence information. According to the amplicon sequence information, the binding sites for the PACE® markers RhK5_8422_105 and RhK5_1295_1946 are located in highly polymorphic regions that contain SNPs and/or INDELs (Supplementary Fig. 3 and Supplementary Fig. 4, Additional File 3), which can lead to difficulties in primer binding for some variants. Additionally, the amplicon primers for the marker RhK5_8422_105 bind not only to the region on chromosome 3 but also to a region on chromosome 1 and amplify a fragment of similar size (10 bp difference). Although the binding sites for the PACE® primer RhK5_10792_6318P showed no polymorphisms (Supplementary Fig. 5, Additional File 3), it is possible that the amplicon primers bind to highly polymorphic regions. This problem can be avoided by developing new amplicons that are not only depending on prior information from SNP chips but are additionally based on published genome sequences of species and cultivars [16, 47, 48] to identify conserved regions for primer binding sites around SNPs. The combination of different reference genomes can reduce the problem of ascertainment bias and errors at problematic sites that occur during assembly due to the highly polymorphic regions in the genome. However, in our study, the amplicons were developed based on PACE® markers so that they could be compared with each other.

### Using our marker set for gene bank management: Identification of mislabelling and duplicate accessions

Mislabelling is a common problem in gene bank management worldwide [11, 12, 49]. It is critical to identify the degree of mislabelling in collections, as this affects the quality of the collection and has negative effects on resource allocation in maintaining the collection. Therefore, the aim of this study was to develop a marker set that effectively discriminates different rose genotypes. This should help in the confirmation of accessions deposited at different locations as well as in the identification of duplicates in the gene bank stock. Of the 94 assumed duplicates located at two different sites, 82 pairs of accessions were confirmed, and eight were refuted using our developed marker set and SSR markers. This is a strong indication of mislabelling given that contamination during the transfer of the samples into the tubes as well as during DNA isolation and downstream applications can be ruled out by re-isolating the DNA. Nevertheless, mixing of the samples may have occurred during harvesting and sample processing, even though the process was performed with the utmost care, including numerous quality control checks such as sampling by three people and transfer of the leaf samples into the reaction tubes by at least two people.

Of the 1,957 accessions analysed with all 18 markers without any failure and complete agreement between the different analysis approaches, 174 accessions grouped into 73 clusters after removing the known expected duplicates. Of these, 58 clusters were subsequently confirmed using five previously published SSR markers and repeated PACE® analyses. As mentioned above, the 15 clusters that showed different band patterns in the SSR analysis, containing 42 accessions, could be the result of a very low remaining error rate due to mixing of the samples during collection. Indeed, for seven groups, confusion of accessions or sampling errors are very likely as according to information from the *Europa Rosarium Sangerhausen* no close relationships between the accessions are known and the phenotypes do not look similar. However, our assessments indicate that the remaining clusters are mostly truly genetically identical accessions. For the accessions in 38 of the clusters, also the phenotypes of the accessions look very similar, and it is very possible that the accessions are identical and have only been given different trade names. This shows how important the information obtained from our marker set in combination with phenotypic comparisons is for gene bank management. However, it is also possible that the accessions are closely related, making it challenging to distinguish these accessions using molecular tools. These clusters might also include cases where sport mutations have been used to register new varieties (known or unknown). In this case, our analyses would not lead to differences in the marker patterns, as we expect only a few genetic changes to cause the sport mutant, and we only covered a very small part of the genome with our markers [50]. Indeed, two of the 59 clusters contained known sport mutants, which we were not able to distinguish. Additionally, one accession and its radiation-induced mutant showed the same marker pattern.

## Conclusion

In our study, we developed a set of 18 robust PACE® markers for the fingerprinting of 4,187 rose accessions. The information provided by this marker set is very useful for gene bank management because it displays a high discriminative capacity. Given that PACE® markers always need to be analysed in larger genotypic groups, the robustness of markers can be improved by the choice of group sizes for allele calling. Mislabelling due to label or location changes is a major problem in gene banks worldwide. In addition, there is occasionally confusion regarding breeding history, and breeders often give the same commercial names to their varieties more than once. With the help of our marker set, the genetic identities can be determined and compared. In future applications, our marker set could be used to develop a core reference set of representative accessions, as recommended by Glaszmann et al. (2010) [51] and Kilian and Graner (2012) [52], and thus optimise the selection of gene bank accessions. Additionally, more than 120 wild rose species were successfully analysed with our marker set; therefore, these 18 PACE® markers might also be useful for future biodiversity studies.

As the price of NGS technologies continues to decrease, future analyses may utilise alternative technologies. Given that only two-thirds of the PACE® markers from our set could be converted into amplicon markers, amplicons independent of the SNP positions of the WagRhSNP chip might represent informative alternatives since more genetic information can be used and so ascertainment bias can be reduced. This should be taken into account when comparing the PACE® genotyping results with the amplicon data. However, the PACE® marker set we have developed is currently more cost- and time-effective for genotyping large rose collections.

## Supplementary Information

Additional file 1: Species names.

Additional file 2: Supplementary Table 1. 20-SNP marker set for genotyping roses.

Additional file 3: Supplementary Figure 1. Distribution of the PACE markers in the genome. Supplementary Figure 2. Theoretical consideration of the required number of markers for tetraploid organisms. Supplementary Figure 3. Binding sites for the PACE marker primers RhK5_8422_105Q. Supplementary Figure 4. Binding sites for the PACE marker primers RhK5_1295_1946P. Supplementary Figure 5. Binding sites for the PACE marker primers RhK5_10792_6318P. 

Additional file 4: Scatterplots from suspected duplicates with one or more SNP differences. 

Additional file 5: Additional File 5. Comparison between PACE^®^ and amplicon scoring within the same SNP analysed in 95 genotypes with a threshold of 50 reads per genotype.

Additional file 6: Additional File 6. Haplotypes and haplotype frequencies for each marker.

Additional file 7: Additional File 7. Haplotype dosages of each genotype.

Additional file 8: Incorrect calling in FitTetra for the marker RhK5_125_737.

## Data Availability

The datasets generated and/or analysed during the current study are available in the figshare repository with the DOIs 10.6084/m9.figshare.25837849 (PACE SNP data), 10.6084/m9.figshare.25846063 (SSR and repeated PACE assay results) and 10.6084/m9.figshare.25836727 (Amplicon sequences). The chip data are available from Wamhoff, D., Patzer, L., Schulz, D. F., Debener, T., & Winkelmann, T. (2023). GWAS of adventitious root formation in roses identifies a putative phosphoinositide phosphatase (SAC9) for marker-assisted selection. Plos one, 18(8), e0287452 under the following DOI: https://doi.org/10.25835/ie9cdzgi. The chip data are available at [17] under following DOI: https://doi.org/10.25835/ie9cdzgi

## Abbreviations

AFLP: Amplified fragment length polymorphism
GBS: Genotyping-by-sequencing
GWAS: Genome Wide Association Studies
RAPD: Random amplified polymorphic DNA
SNP: Single nucleotide polyporphisms
SSR: Simple sequence repeats
