# Supplementary material for: Development of a robust SNP marker set for genotyping diverse gene bank collections of polyploid roses
Source: BMC Plant Biol. 2024 Nov 14;24:1076. doi: 10.1186/s12870-024-05782-2 (PMC11562693; doi:10.1186/s12870-024-05782-2)
Supplement: Supplementary file 3 — Additional file 3: Supplementary Figure 1. Distribution of the PACE markers in the genome. Supplementary Figure 2. Theoretical consideration of the required number of markers for tetraploid organisms. Supplementary Figure 3. Binding sites for the PACE marker primers RhK5_8422_105Q. Supplementary Figure 4. Binding sites for the PACE marker primers RhK5_1295_1946P. Supplementary Figure 5. Binding sites for the PACE marker primers RhK5_10792_6318P. [file 12870_2024_5782_MOESM3_ESM.docx]

**Additional file for:**

**Development of a robust SNP marker set for genotyping diverse gene bank collections of polyploid roses**

Laurine Patzer, Tim Thomsen, David Wamhoff, Dietmar Frank Schulz, Marcus Linde, Thomas Debener


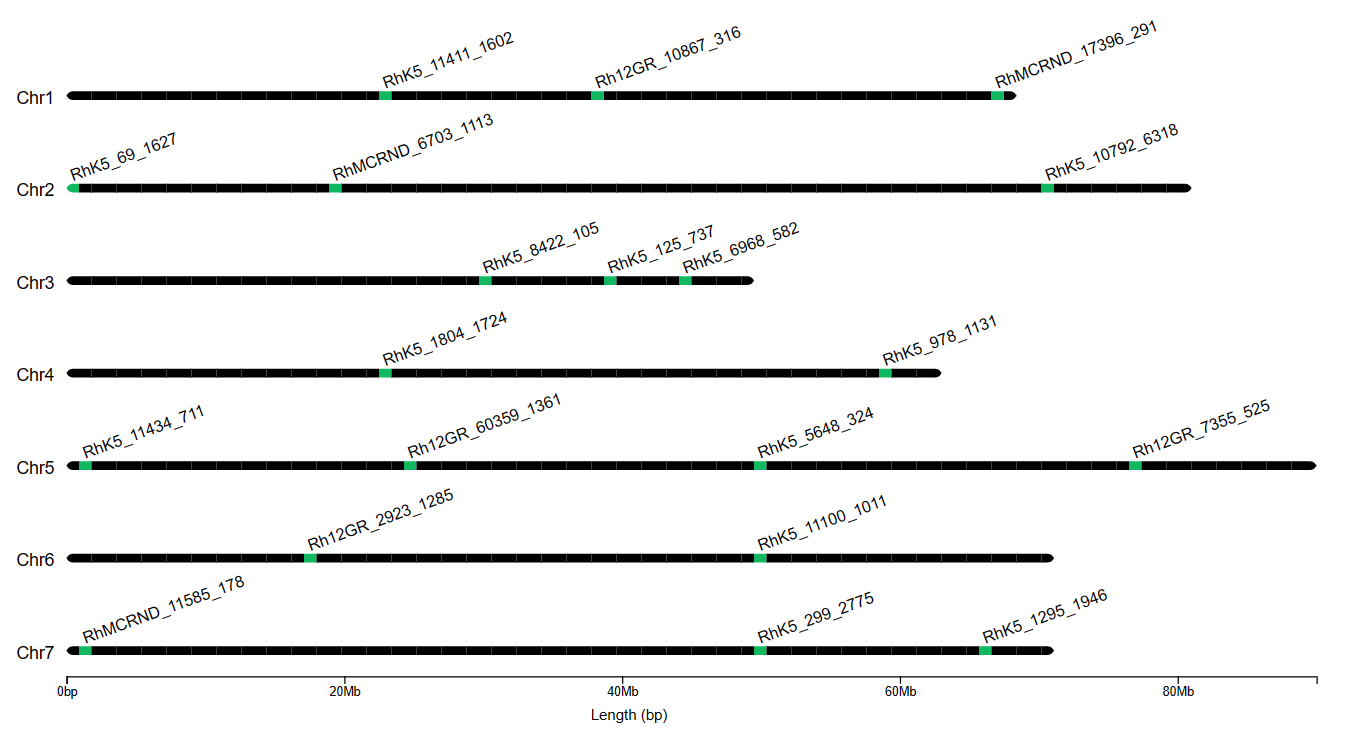


**Supplementary Figure 1. Distribution of the PACE markers in the genome.**

The seven chromosomes of the rose genome are shown. The green stripes represent the location of the respective marker from the marker set. Rosa chinensis Genome V1.0 was used to map the markers in the genome. The graphic was created with ChromoMap.


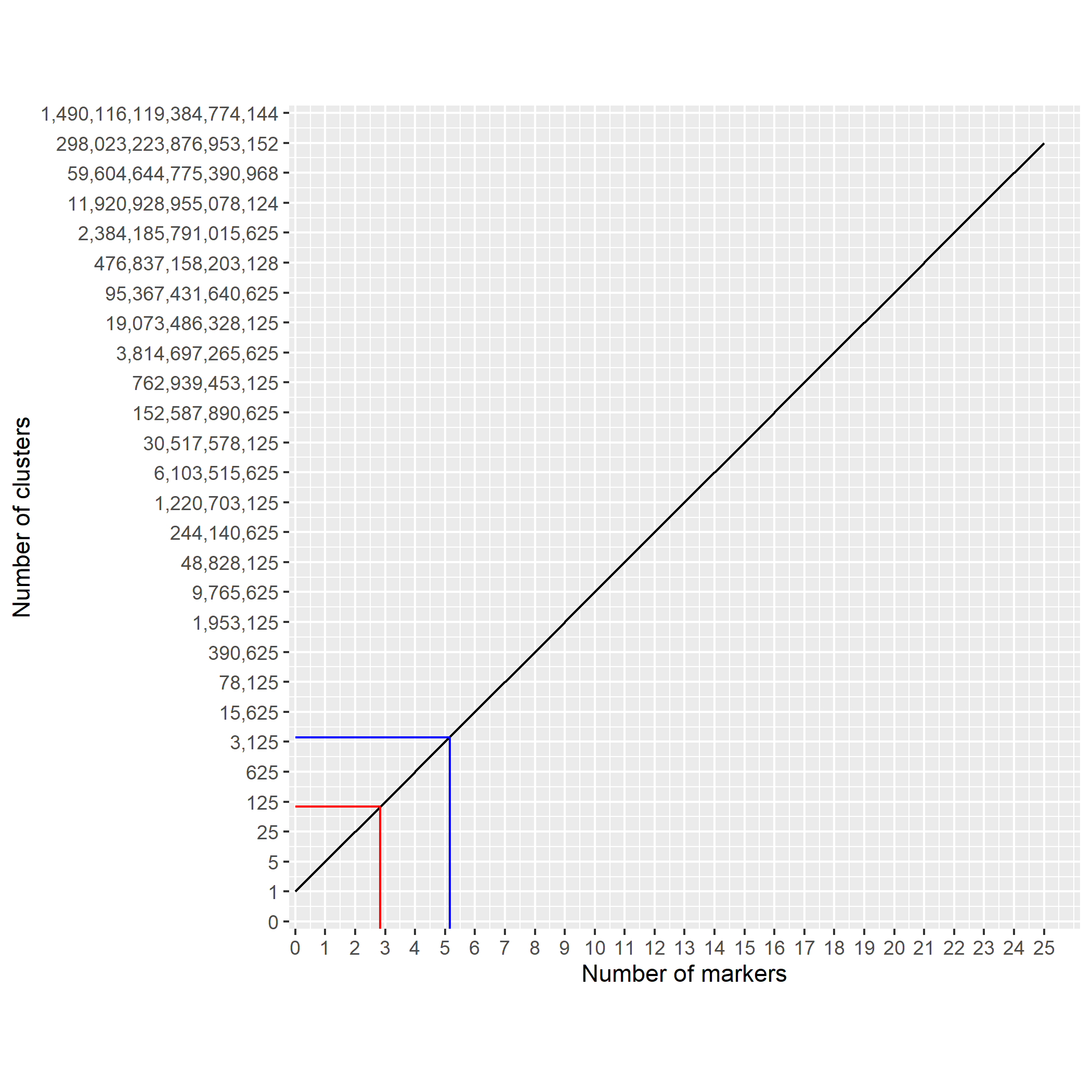


**Supplementary Figure 2. Theoretical consideration of the required number of markers for tetraploid organisms**.

The red line indicates the required number of markers for distinguishing 96 different genotypes, the blue line indicates the number of markers for distinguishing 4000 genotypes.


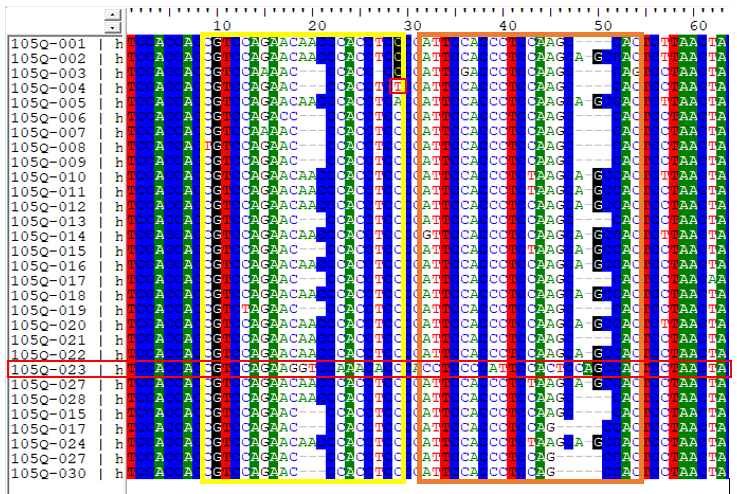


**Supplementary Figure 3. Binding sites for the PACE marker primers RhK5_8422_105Q.**

Orange: Binding site for reverse primer (GTGGCTGCTTGGAGGGTGGAAT), yellow: Binding site for Forward primers (CGTCCAGAACAACCCACCTC[A/C]). Sequence 105Q-023 and not expected “T” Allele in the PACE SNP region are highlighted in red.


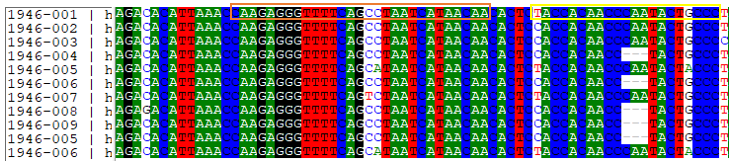


**Supplementary Figure 4. Binding sites for the PACE marker primers RhK5_1295_1946P.**

*Orange: Binding site for reverse primer (CAAGAGGGTTTTCAGCCTAATCATAACAA), yellow: Binding site for forward primers (TAGGGCAGTATTGGGTTGTGGT[A/G]).*


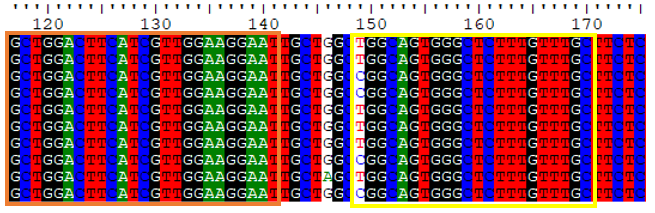


**Supplementary Figure 5. Binding sites for the PACE marker primers RhK5_10792_6318P.**

*Orange: Binding site for reverse primer (GCTGGACTTCATCGTTGGAAGGAAT), yellow: Binding site for forward primers (GCAAACAAAGAGCCCACTGCC[A/G]).*
