# Supplementary material for: Development of a robust SNP marker set for genotyping diverse gene bank collections of polyploid roses
Source: BMC Plant Biol. 2024 Nov 14;24:1076. doi: 10.1186/s12870-024-05782-2 (PMC11562693; doi:10.1186/s12870-024-05782-2)

**Additional File 4. Scatterplots from suspected duplicates with one or more SNP differences.**

*Scatterplots are created based on fluorescence signals from the PACE Assay. Genotyping was performed with FitTetra. “geno” = Allele dosage group. Samples are located on different plates.*

**Samples with 1 SNP difference from verification of suspected duplicates.**


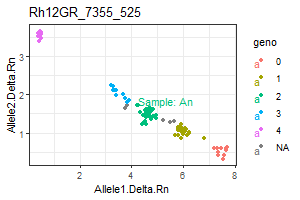

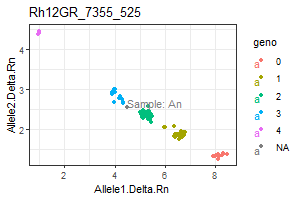

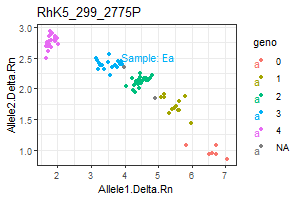

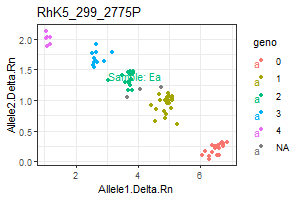

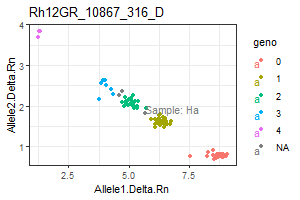

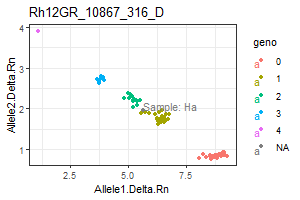

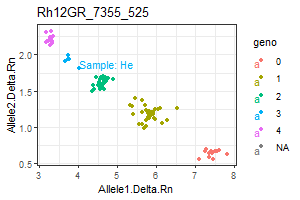

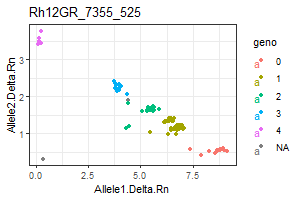

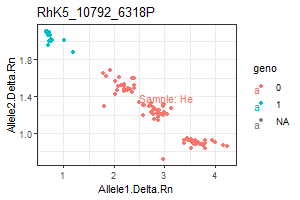

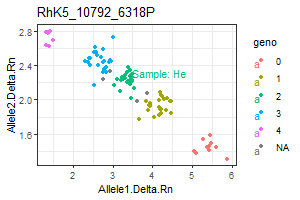

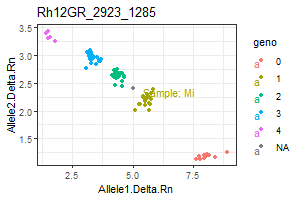

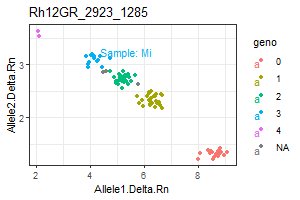

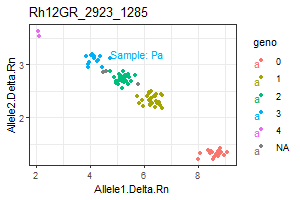

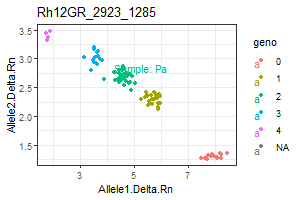

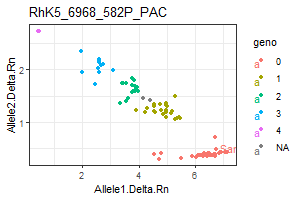

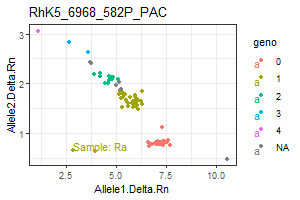

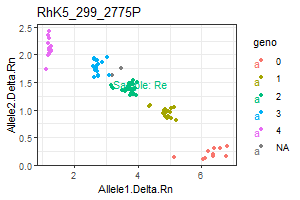

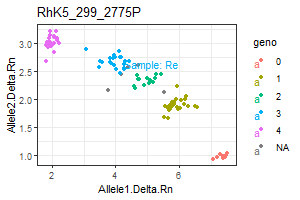

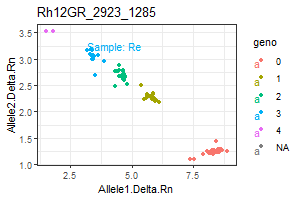

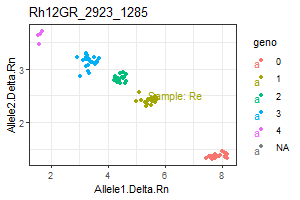

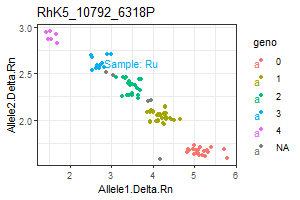

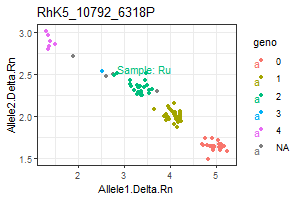

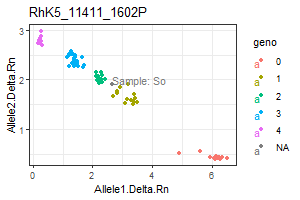

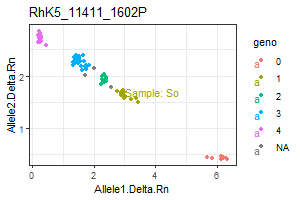


**Samples with 2 SNPs difference from verification of suspected duplicates.**


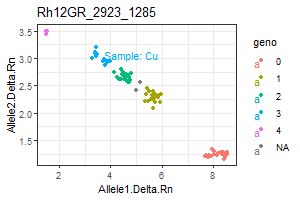

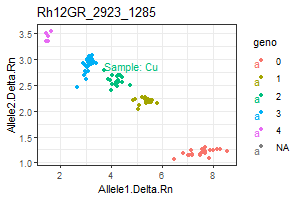

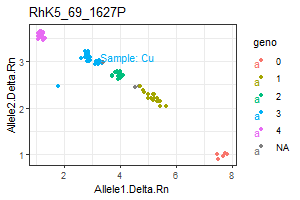

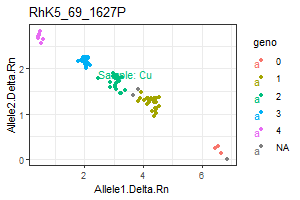

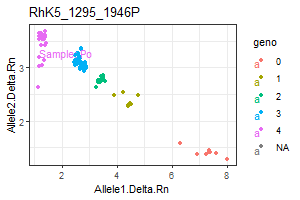

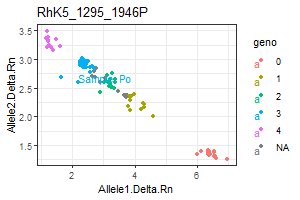

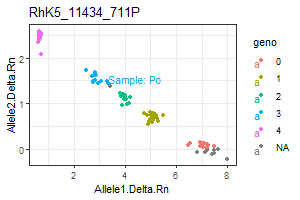

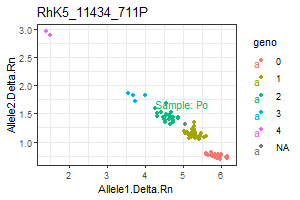


**Samples with 3 SNPs difference from verification of suspected duplicates.**


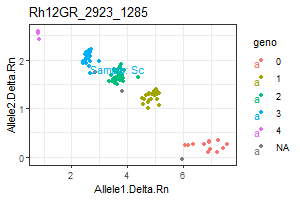

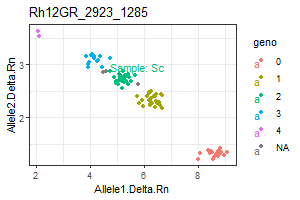

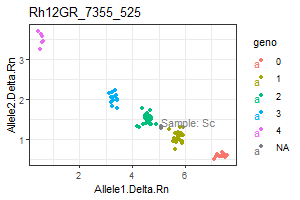

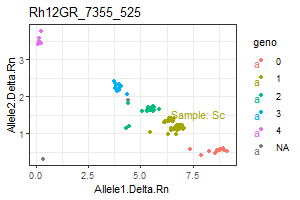


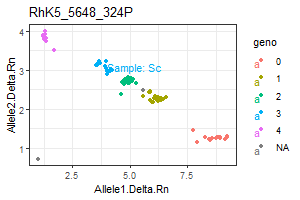


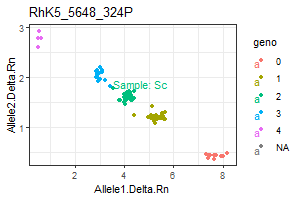

Supplement: Supplementary file 4 — Additional file 4: Scatterplots from suspected duplicates with one or more SNP differences. [file 12870_2024_5782_MOESM4_ESM.docx]
