# Supplementary material for: Development of a robust SNP marker set for genotyping diverse gene bank collections of polyploid roses
Source: BMC Plant Biol. 2024 Nov 14;24:1076. doi: 10.1186/s12870-024-05782-2 (PMC11562693; doi:10.1186/s12870-024-05782-2)
Supplement: Supplementary file 5 — Additional file 5: Additional File 5. Comparison between PACE® and amplicon scoring within the same SNP analysed in 95 genotypes with a threshold of 50 reads per genotype. [file 12870_2024_5782_MOESM5_ESM.docx]

**Additional File 5. Comparison between PACE^®^ and amplicon scoring within the same SNP analysed in 95 genotypes with a threshold of 50 reads per genotype.**

| **Marker** | **Number of genotypes with identical scoring** | **Number of genotypes with differences in scoring** | **Number of unscored genotypes in total** | **Number of unscored genotypes in the PACE assay** | **Number of unscored genotypes in the amplicon analysis** |
| --- | --- | --- | --- | --- | --- |
| Rh12GR_2923_1285 | 82 | 0 | 13 | 5 | 10 |
| RhK5_11411_1602 | 89 | 1 | 5 | 2 | 4 |
| RhK5_978_1131 | 84 | 1 | 10 | 3 | 9 |
| RhMCRND_11585_178 | 68 | 1 | 26 | 4 | 24 |
| RhMCRND_6703_1113 | 78 | 1 | 16 | 13 | 5 |
| RhK5_6968_582 | 80 | 2 | 13 | 5 | 10 |
| RhK5_299_2775 | 76 | 3 | 16 | 2 | 15 |
| Rh12GR_7355_525 | 66 | 4 | 25 | 13 | 17 |
| RhK5_69_1627 | 67 | 9 | 19 | 6 | 17 |
| RhK5_5648_324 | 74 | 16 | 5 | 1 | 5 |
| RhK5_10792_6318 | 66 (55) | 19 (27) | 10 (13) | 1 (1) | 10 (13) |
| RhK5_8422_105 | 63 | 22 | 10 | 3 | 9 |
| RhK5_1295_1946 | 35 (34) | 41 (43) | 19 (18) | 5 (5) | 17 (16) |

The numbers in brackets are the numbers in the repetition.
