# Supplementary figures and images for: Development of a robust SNP marker set for genotyping diverse gene bank collections of polyploid roses
Source: BMC Plant Biol. 2024 Nov 14;24:1076. doi: 10.1186/s12870-024-05782-2 (PMC11562693; doi:10.1186/s12870-024-05782-2)

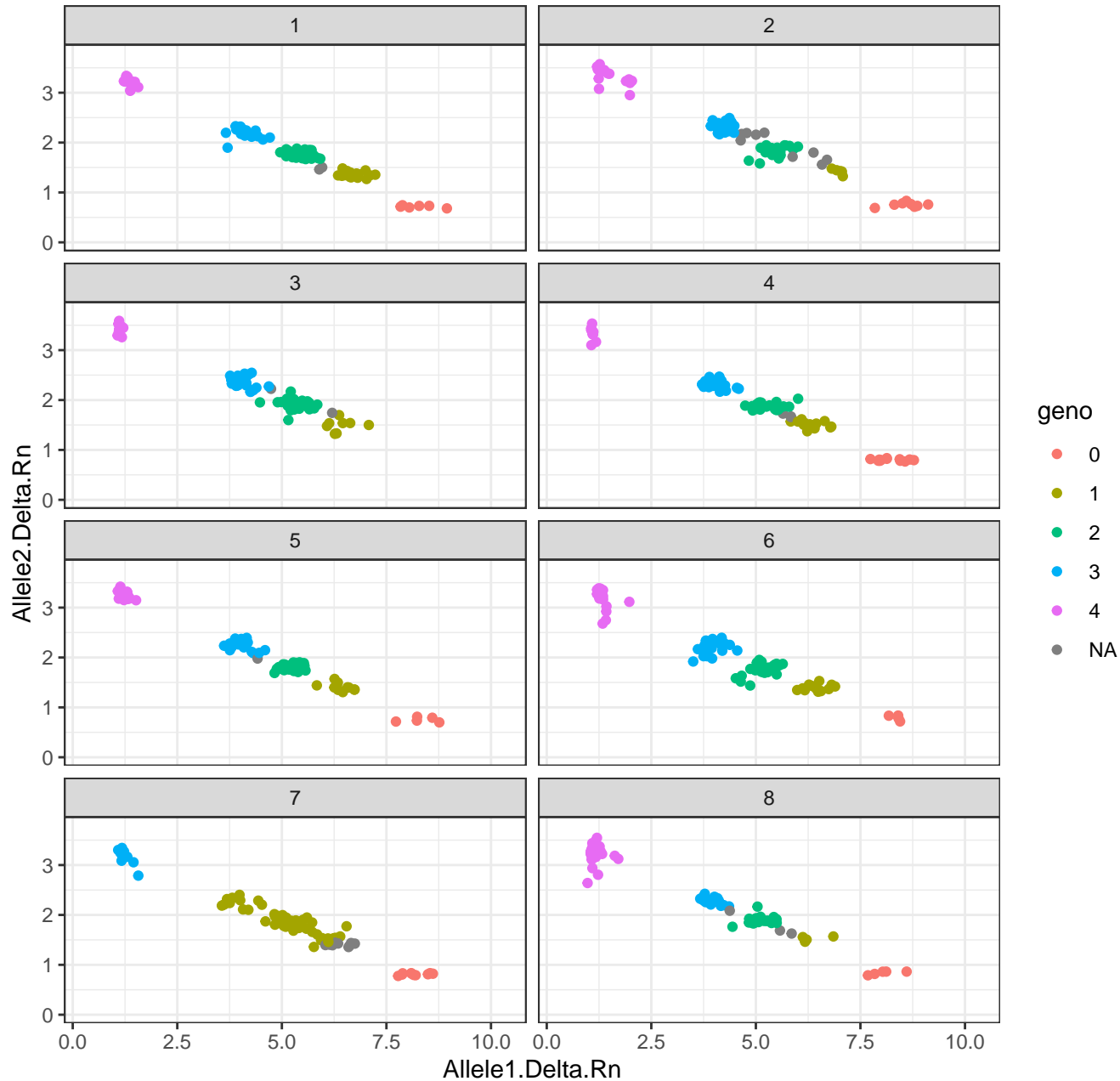

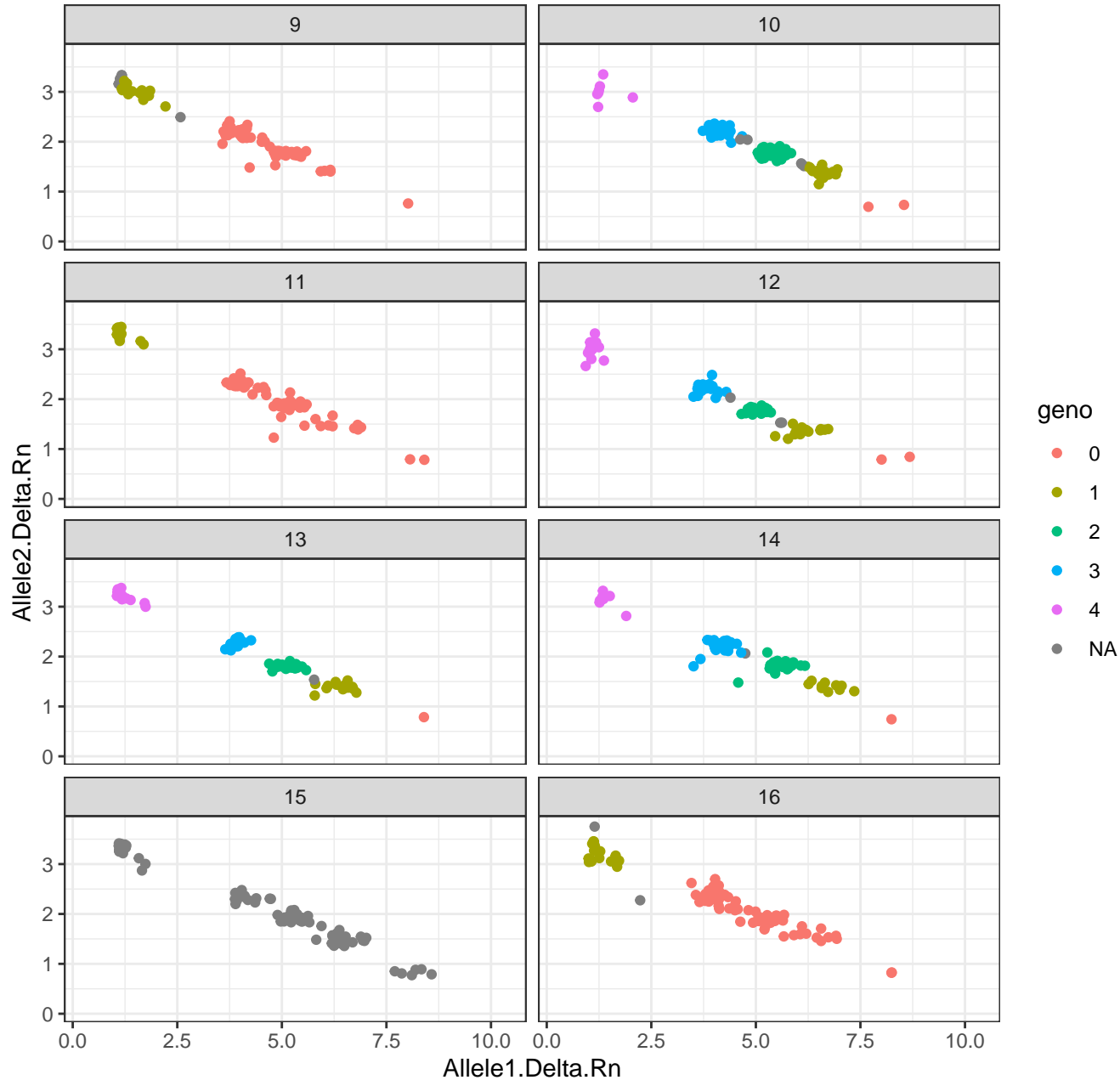

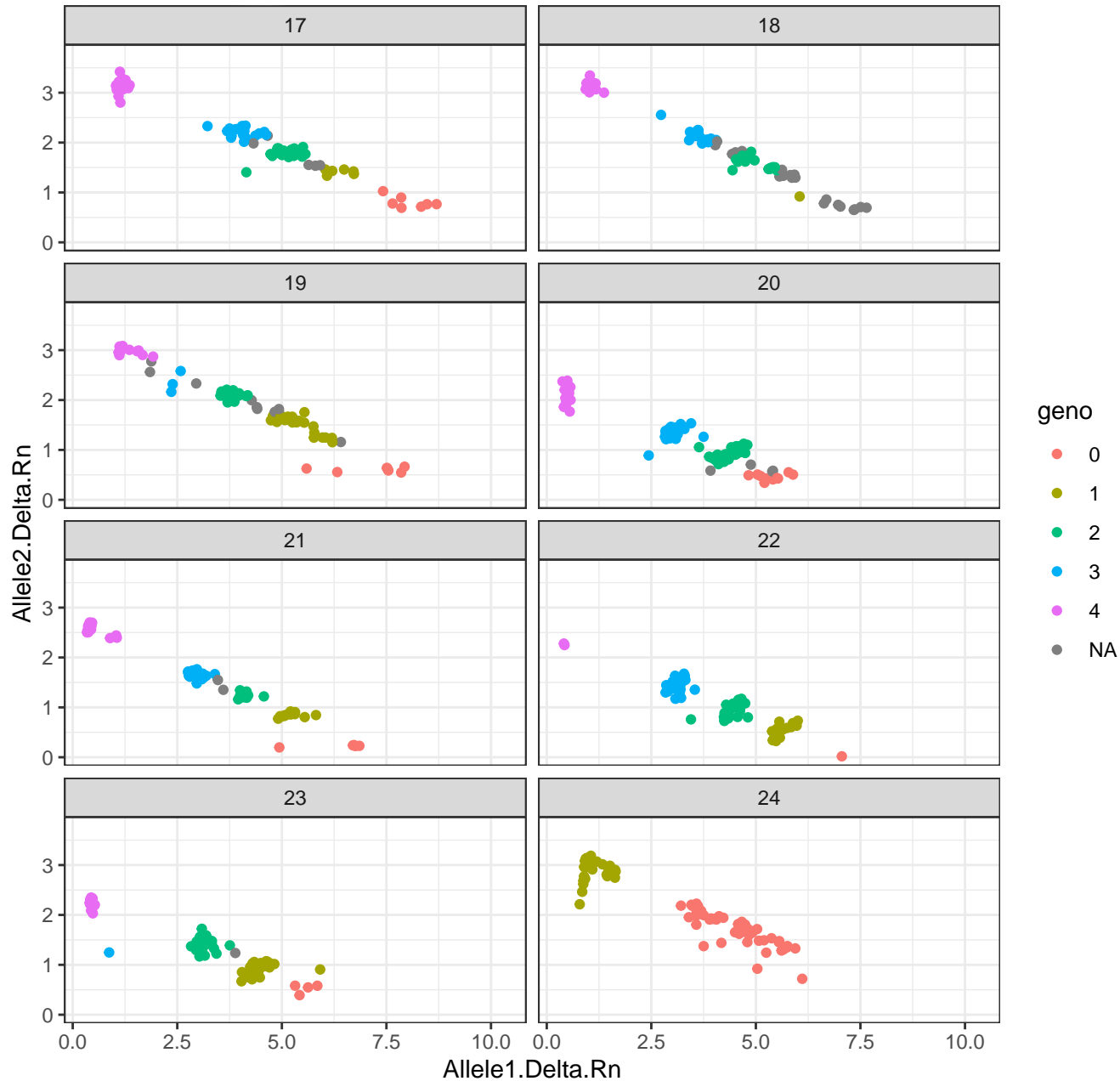

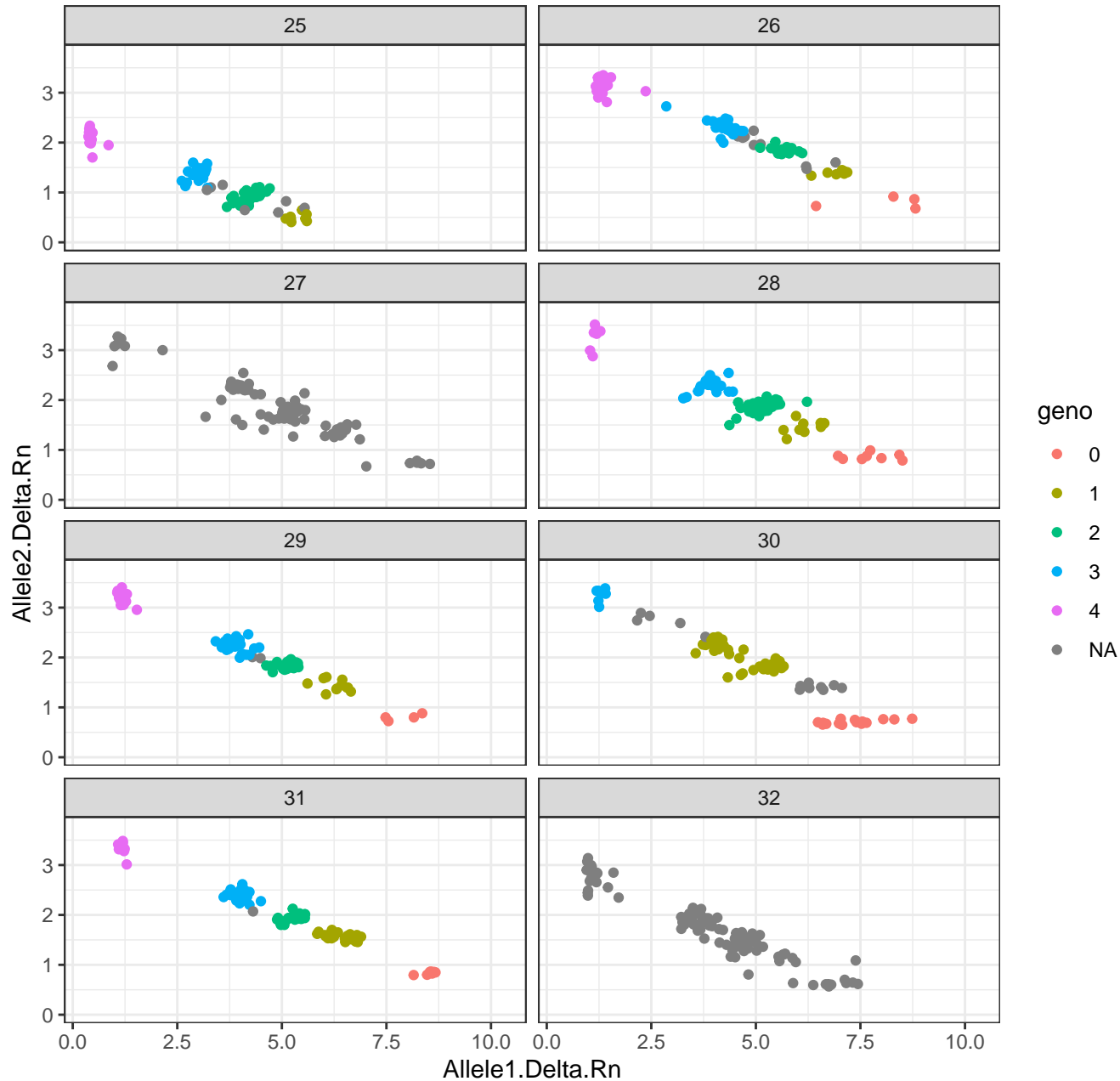

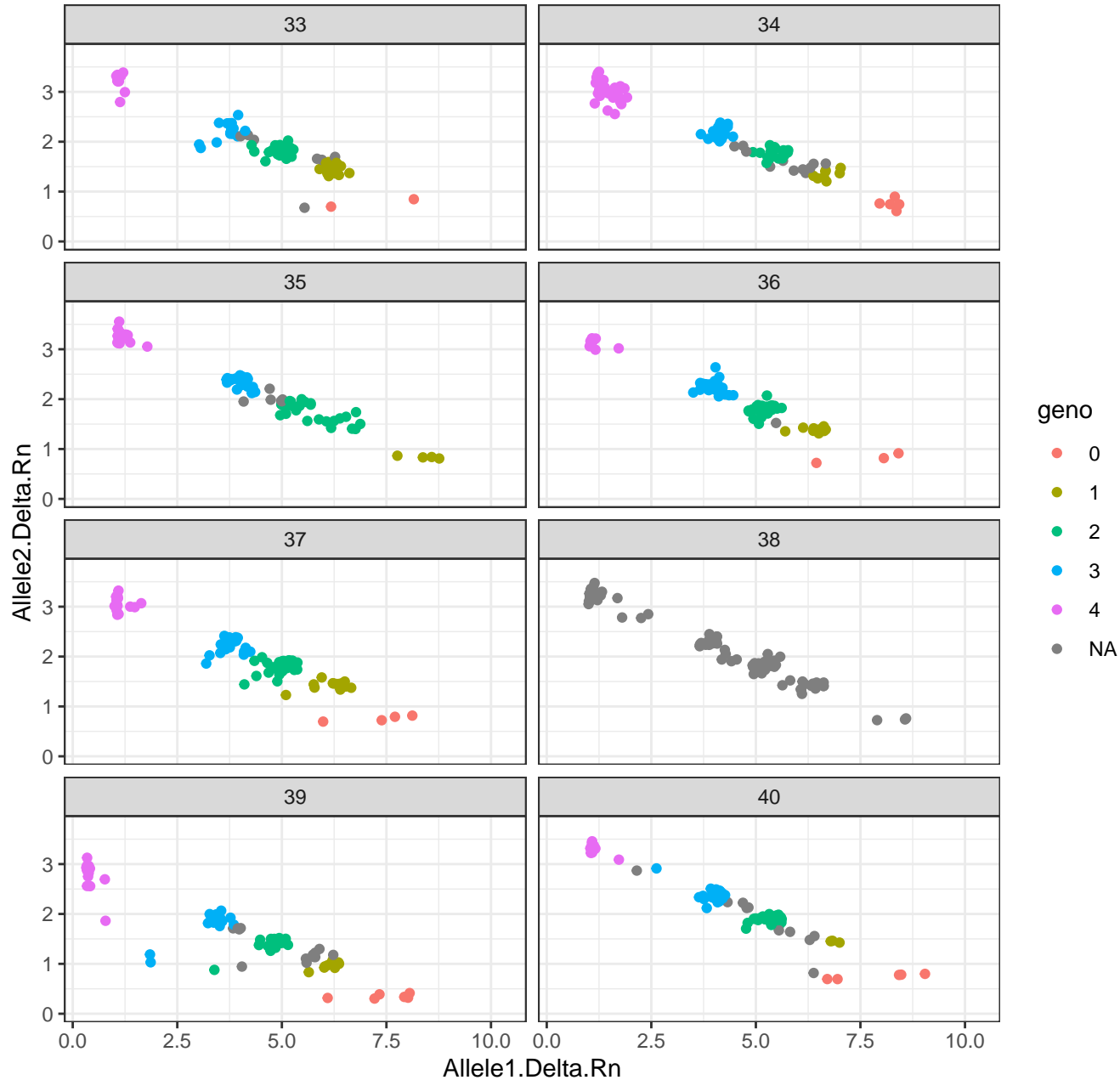

Allele2.Delta.Rn

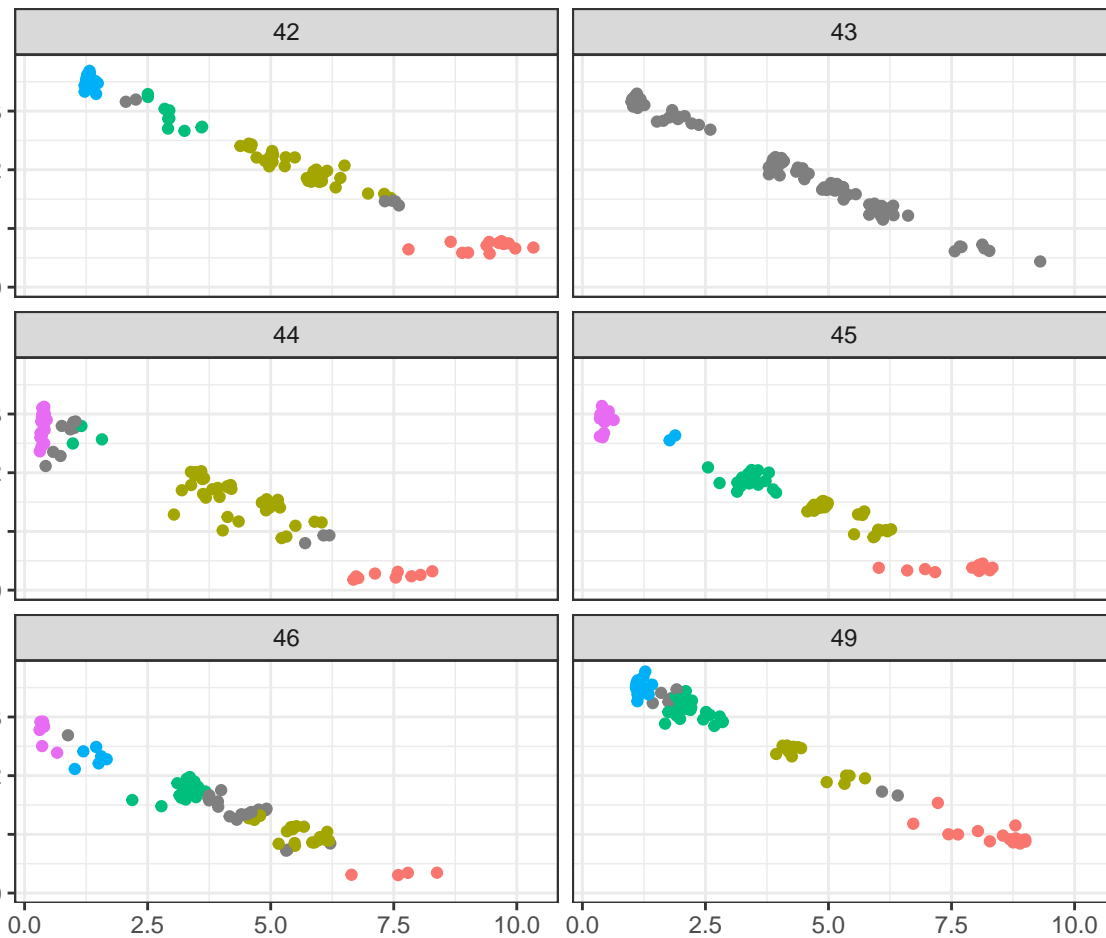

geno

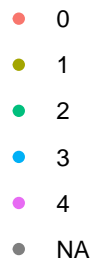

Allele1.Delta.Rn

Supplement: Supplementary file 8 — Additional file 8: Incorrect calling in FitTetra for the marker RhK5_125_737. [file 12870_2024_5782_MOESM8_ESM.pdf]
